# Why Alcohol Intoxicates

**Published:** 1885-06

**Authors:** 


### HALL'S JOURNAL OF HEALTH

Vol. 32. JUNE, 1885. No. 6.

### Why Alcohol Intoxicates.

Those of us who are unaccustomed to strong drink have often noticed how quickly a glass of wine or a small amount of distilled liquor "goes to the head."

Most of us know that this effect is caused by the direct presence of alcohol in the blood, but it is not generally known just how it gets there.

To explain the delicate but simple operation of conveying the alcohol into the whole system is the object of this article.

All liquors, wine and beer, are merely alcohol, diluted with water and flavored by the juices of the fruit or grain from which the drink is made. The beverage, being taken into the stomach, comes in contact with the lining of that organ.

Now, this lining is provided with a network of delicate blood vessels, which are very small and have a thin membranous covering. Alcohol has the property of permeating this coating and being taken up at once by the blood within the capillaries, which carries it away to other parts of the system. Water, however, requires a much longer time to be absorbed, and as the alcohol becomes partially removed from the contents of the stomach, they pass into the small intestines. A small percentage of the alcohol which remains after this takes place, is rapidly taken up by the lacteals or the absorbent vessels of the small intestine and enters the main blood stream by way o the thorasic duct. The alcohol all eventually goes to the heart and thence through the liver into the general circulation.

All the organs in which blood circulates are now brought into contact with the mixture of blood and alcohol.

The nerve pulp, the brain substance and the great nerve centres are rich in blood vessels, and being the most sensitive part of the body to the action of alcohol, by reason of the fact that the natural moisture of the nerves, on which they largely depend for healthy action is largely taken up by the alcohol and conveyed to the blood, they soon lose their control of the muscles, both voluntary and involuntary.

The heart, as a consequence, beats more rapidly, having less resistance to meet. The muscles of the veins and arteries relax, and the capillaries expand.

A feeling of warmth and flushing of the face is the result. The brain acts more quickly and thought and speech flows more freely.

Upon taking a still greater quantity of alcohol, some of the functions which are governed by the spinal cord become completely narcotized. The legs, feet and lips are first to feel this effect.

As more and more alcohol is taken, its effect progresses from one nerve to another, until the brain itself is stupefied and the mind is totally under the deadly influence, while the man sinks himself to the lowest level of mere animal existence. Finally, real temporary paralysis of all the nerve centres sets in, consciousness is lost, and the victim sinks into a sleep. The beating of the heart and the moving of the lungs, is all that distinguishes him from the clay from which he came.

Sense, reason, mind, all gone. What can be lower or more degraded?

